# Immune Metabolism in TH2 Responses: New Opportunities to Improve Allergy Treatment — Disease-Specific Findings (Part 1)

**DOI:** 10.1007/s11882-022-01057-8

**Published:** 2022-11-28

**Authors:** A. Goretzki, J. Zimmermann, H. Rainer, Y.-J. Lin, Stefan Schülke

**Affiliations:** Vice President’s Research Group 1: Molecular Allergology, Paul-Ehrlich-Institut, Paul-Ehrlich-Str. 51-59, 63225 Langen, Germany

**Keywords:** Allergy, Immune metabolism, Immunotherapy, Asthma, Food allergy, Allergic dermatitis

## Abstract

**Purpose of Review:**

Recent high-level publications have shown an intricate connection between immune effector function and the metabolic state of the respective cells. In the last years, studies have begun analyzing the metabolic changes associated with allergies. As the first part of a two-article series, this review will briefly summarize the basics of immune metabolism and then focus on the recently published studies on metabolic changes observed in allergic patients.

**Recent Findings:**

In the last 3 years, immune-metabolic research in allergology had a clear focus on asthma with some studies also reporting findings in food allergy and atopic dermatitis. Current results suggest asthma to be associated with a shift in cellular metabolism towards increased aerobic glycolysis (Warburg metabolism), while also displaying substantial changes in fatty acid- and amino acid metabolism (depending on investigated patient collective, asthma phenotype, and disease severity).

**Summary:**

Understanding immune-metabolic changes in allergies will allow us to (I) better understand allergic disease pathology and (II) modulate immune-metabolic pathways to improve allergy treatment.

## Introduction

Recent high-level publications have shown immune effector function and therefore, the overall induced immune responses mediated by different immune cell types to be closely connected to the respective cells’ metabolism [1]. Here, immune cell metabolism not only provides the energy needed to sustain the demands of the activated and highly active cells but also strongly contributes to the production of important immune effector molecules (reviewed in [2, 3]). While we have started to understand the principles of immune cell metabolism and its connection to the respective cells’ effector function, further understanding the metabolic changes associated with immune cell activation will allow us to (I) better understand allergic disease pathology and (II) modulate immune-metabolic pathways to improve treatment of allergic diseases.

As part one of a two-article series, this review will briefly summarize the basics of immune metabolism and then focus on the recently published studies investigating the immune-metabolic changes observed in allergic patients. Part two will go on to summarize recent findings in distinct cell types important for the establishment and maintenance of allergic reactions and finally describe the initial application of these findings for the treatment of allergic diseases.

## Short Introduction into the Basics of Immune Metabolism

### Basic Cellular Metabolism

Metabolism is a very complex network of biochemical reactions that aims at providing the cell with exactly the right molecules at the right time. Put simply, cells break down glucose, fatty acids, and amino acids to generate both energy (in the form of ATP) and metabolites that are used as the building blocks of other cellular pathways and components.

**Fig. 1:**
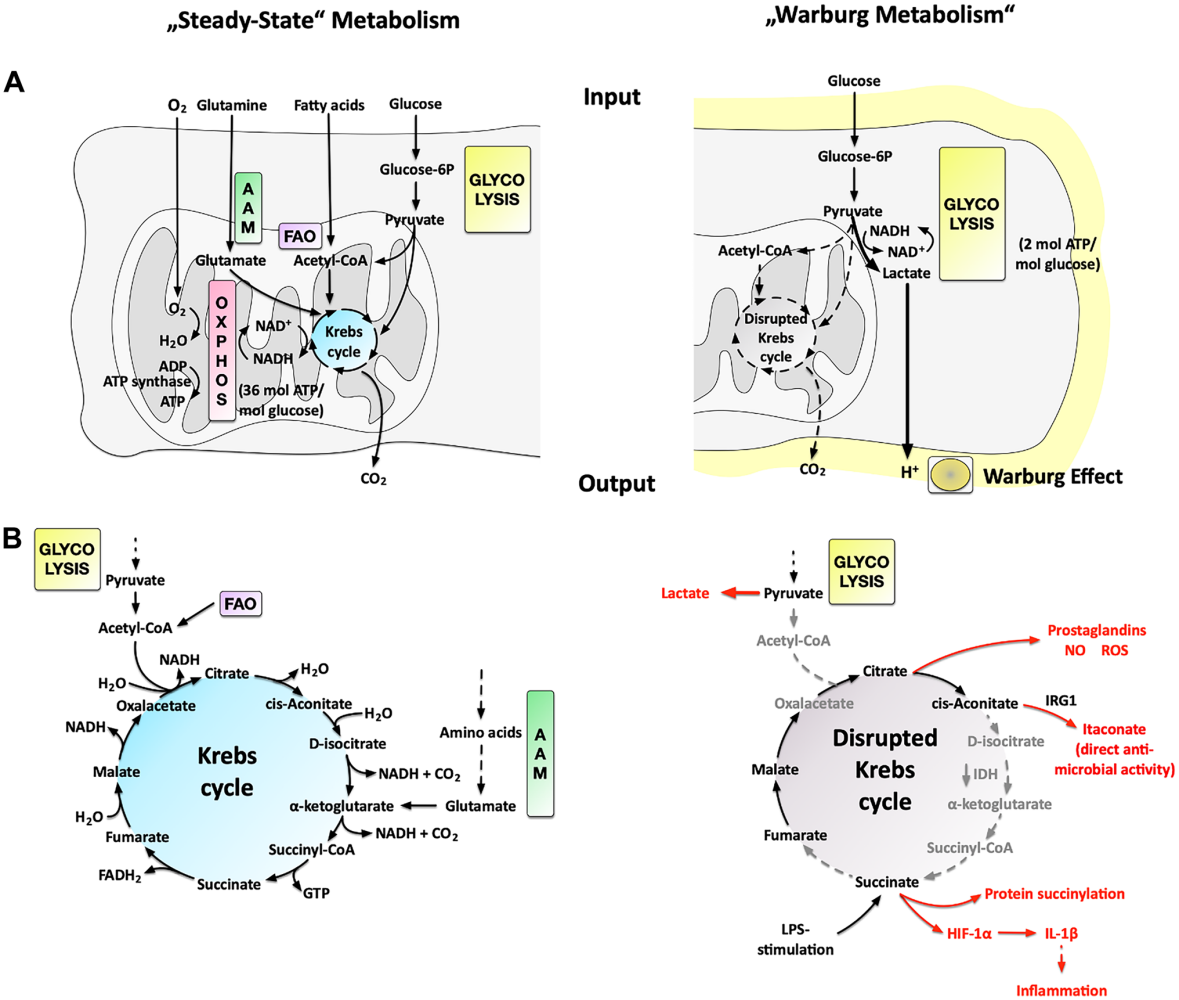
Comparison of steady-state cellular metabolism to metabolic adaptations observed in activated DCs and macrophages. Under normal conditions (**A**, left), cells take up glucose from their medium to generate pyruvate via glycolysis in their cytosol. Pyruvate is subsequently imported into the mitochondrion and further oxidized to CO_2_ via the multi-step Krebs cycle to generate reduction equivalents (NADH & FADH_2_, **B**, left). These reduction equivalents are then transferred in oxidative phosphorylation (OxPhos) to oxygen as a terminal electron acceptor to generate a proton gradient over the inner mitochondrial membrane. This protein gradient drives the generation of ATP via the ATP synthase complex (**A**, left). The Krebs cycle can also be fueled by either fatty acids degraded into acetyl-CoA molecules during fatty acid oxidation (FAO) or glutamate derived from amino acid metabolism (AAM). Under certain conditions, cancer cells, strongly proliferating cells, or activated immune cells switch to predominantly producing lactate from glucose instead of fully oxidizing glucose into CO_2_ in the mitochondrion (**A**, right). In addition, in activated immune cells, the lack of pyruvate results in a “disrupted” Krebs cycle which is used for the generation of important biosynthetic intermediates that contribute to the cell’s effector function (**B**, right). Here, directly and indirectly antimicrobial molecules such as prostaglandins, NO, ROS, or itaconate are generated from the disruption of the Krebs cycle at cis-aconitate. In addition, the Krebs cycle can also be disrupted at succinate (e.g., shown for LPS-stimulated macrophages). Here, the accumulating succinate is sensed by the cell as a danger signal that drives pro-inflammatory responses via both stabilization of HIF-1α and post-translational protein succinylation. Molecules contributing to the effector function of the activated immune cells are indicated in red. Abbreviations: ROS, reactive oxygen species; NO, nitric oxide; IRG1, immune-responsive gene 1; HIF-1α, hypoxia-inducible factor 1α

Glucose, either taken up from the respective cell surrounding via specialized transporters or generated intracellularly via the enzymatic mobilization of glycogen molecules, is broken down in a multi-step process of 11 serial enzymatic reactions called *glycolysis*, resulting in the cleavage of the C6 body glucose into two C3 bodies pyruvate and two molecules of ATP (Fig. 1A, left) [4].

The generated pyruvate is transported into the mitochondrion, where it is further oxidized into CO_2_ in a multi-step cycle of nine enzymatic reactions that are termed the *Krebs cycle* (Fig. 1B, left). The Krebs cycle can also be replenished at specific points by either acetyl-CoA generated from fatty acids during *fatty acid oxidation* or by glutamate generated in *amino acid metabolism* (Fig. 1A, left).

Within the reactions of the Krebs cycle, a part of the energy released from fully oxidizing the carbon atoms of pyruvate is stored in the form of the reduction equivalents NADH and FADH_2_. As a next step, in a process termed *oxidative phosphorylation* (OxPhos), the electrons are stripped off NADH and FADH_2_ within the mitochondria and are transported stepwise over the mitochondrial complexes I to IV, located at the inner mitochondrial membrane, where they are finally transferred to oxygen as terminal electron acceptor (resulting in the formation of water) [5].

This *electron transport chain* (ETC) is used to build up a proton gradient over the inner mitochondrial membrane that allows for the generation of large amounts of ATP via the ATP synthase complex. Leveraging this complex series of reactions, up to 36 molecules of ATP can be generated from one molecule of glucose [6].

Under certain conditions, the pyruvate generated in glycolysis can also be metabolized into lactate (Fig. 1A, right). This process, which is constitutively used by certain bacteria and yeasts to generate ethanol, is energetically less efficient (2 molecules of ATP generated in glycolysis vs. up to 36 molecules generated in OxPhos) as it entails only a partial oxidation of the carbon atoms contained within glucose but allows for a faster generation of energy and is independent of oxygen availability [6]. Consequently, it is used by quickly dividing cancer cells that are often not connected to ample oxygen supply as well as certain highly proliferative cells and activated immune cells [7, 8]. As this process was first described in the early twentieth century by Otto Warburg in cancer cells [9], it is commonly referred to as either “Warburg Effect/Metabolism” or aerobic glycolysis as these cells display a high rate of glycolysis even in the presence of oxygen [10].

### Cellular Metabolic Adaptations Observed in Activated Immune Cells

Before reviewing the newly published results of the last years, we will shortly summarize the high-level immune-metabolic adaptations observed in activated immune cells (reviewed in more detail in [11]).

With their ability to take up, process, and present antigens, antigen presenting cells (APCs) are critical in bridging innate and adaptive immune responses. Already early on, dendritic cells (DCs) and macrophages, the two most important types of APCs, were shown to undergo distinct metabolic changes upon activation switching their metabolism towards enhanced glycolysis, resulting in a “disrupted” Krebs cycle [12–16] (Fig. 1B, right). This rewiring of DC and macrophage metabolism was shown to be of critical importance for their particular effector functions, such as cell migration [17], phagocytosis [18], production of pro-inflammatory cytokines [19], and CD4^+^ and CD8^+^ T-cell activation [20].

Moreover, a conserved switch of innate immune cells towards a predominant production of energy via aerobic glycolysis is an adaptation of these cells to function in the often highly hypoxic conditions of acutely inflamed tissues [21–23]. Mechanistically, the hypoxia-inducible factor 1α (HIF-1α) was found to be the molecular link between oxygen deprivation in inflamed tissues and immune cell function with HIF-1α being critically important for both maintaining energy homeostasis and mediating the activation and effector function of myeloid cells [24].

Besides allowing immune cells to function in acutely inflamed hypoxic or even oxygen-free tissues, the switch towards a predominantly glycolytic metabolism also has another major consequence for these cells. The preferential metabolization of glucose to lactate also leads to a “disrupted” Krebs cycle, resulting from the undersupply of the mitochondria with pyruvate (which is metabolized into lactate to regenerate the limited cytosolic NADH pool in order to maintain glycolytic flux) [25]. Under these conditions, the Krebs cycle is arrested at defined points, which results in the accumulation of certain intermediary molecules that are in turn used as substrates to generate highly important effector molecules (Fig. 1B, right) [3]. In LPS-stimulated macrophages, these breaking points were reported to be located after citrate and after succinate (Fig. 1B, right) [19, 26].

Here, the accumulation of citrate, cis-aconitate, and D-isocitrate (due to a decreased expression of isocitrate dehydrogenase (IDH), which normally converts D-isocitrate to α-ketoglutarate [26]) allows for the generation of both directly and indirectly antimicrobial molecules such as prostaglandins, nitric oxide (NO), reactive oxygen species (ROS), or itaconate (Fig. 1B, right, reviewed in [25]). In addition, succinate accumulating in LPS-stimulated macrophages was shown to promote glycolysis and IL-1β production via the stabilization of HIF-1α as well as acting as a substrate for post-translational protein succinylation (Fig. 1B, right, reviewed in [3]).

In contrast to the highly glycolytic DCs and macrophages, other innate immune cells were shown to either display metabolic flexibility (employing both glycolysis and OxPhos, e.g., eosinophils or mast cells) [27–29] or to mainly rely on fatty acid oxidation (FAO) (e.g., ILC2s) [30].

ILC2s need to fulfill their effector function at locally nutrient (and glucose)-deprived environments created by parasites at the respective sites of infection [31]. Therefore, their reported fatty acid dependency may be the evolutionary consequence of having to function in this special niche.

Regarding cells of the adaptive immune system, the reported metabolic phenotypes are more complex: while acutely activated effector T cells generally speaking show high rates of mammalian target of rapamycin (mTOR)-dependent glycolysis, regulatory T cells, and long-lived memory T cells mainly rely on AMP-activating protein kinase-mediated increased FAO [32, 33].

Finally, while IL-4-activated B cells in allergic contexts employ both aerobic glycolysis and FAO to fulfill their energetic demands and effector functions (reviewed in [34]), highly proliferative B cells in germinal centers were shown to mainly generate their required energy via FAO, but not glycolysis [35].

After establishing the basics of immune metabolism, we will review the disease- and cell type-specific findings as well as novel strategies to improve the treatment of allergic diseases by targeting immune cell metabolism.

## Disease-Specific Findings in Allergy

In the last 3 years, immune-metabolic research in allergology had a clear focus on asthma with some studies also reporting findings in food allergy and atopic dermatitis.

### Asthma

Asthma is a complex disease comprising different phenotypes that are characterized by airway-inflammation, -remodeling, and -hyperreactivity. Studies have repeatedly shown the presence of metabolic anomalies in asthmatic patients and many metabolic pathways, including glycolysis [36–40], amino acid metabolism [41, 42], fatty acid metabolism [43, 44], and sphingosine metabolism [45–47], have been reported to be associated with asthma. Especially obese asthma, which trends towards higher disease severity, is associated with increases in glycolysis and both basal and maximal respiration in airway epithelial cells and platelets [48], as well as overall increased oxidative stress [48, 49].

**Fig. 2:**
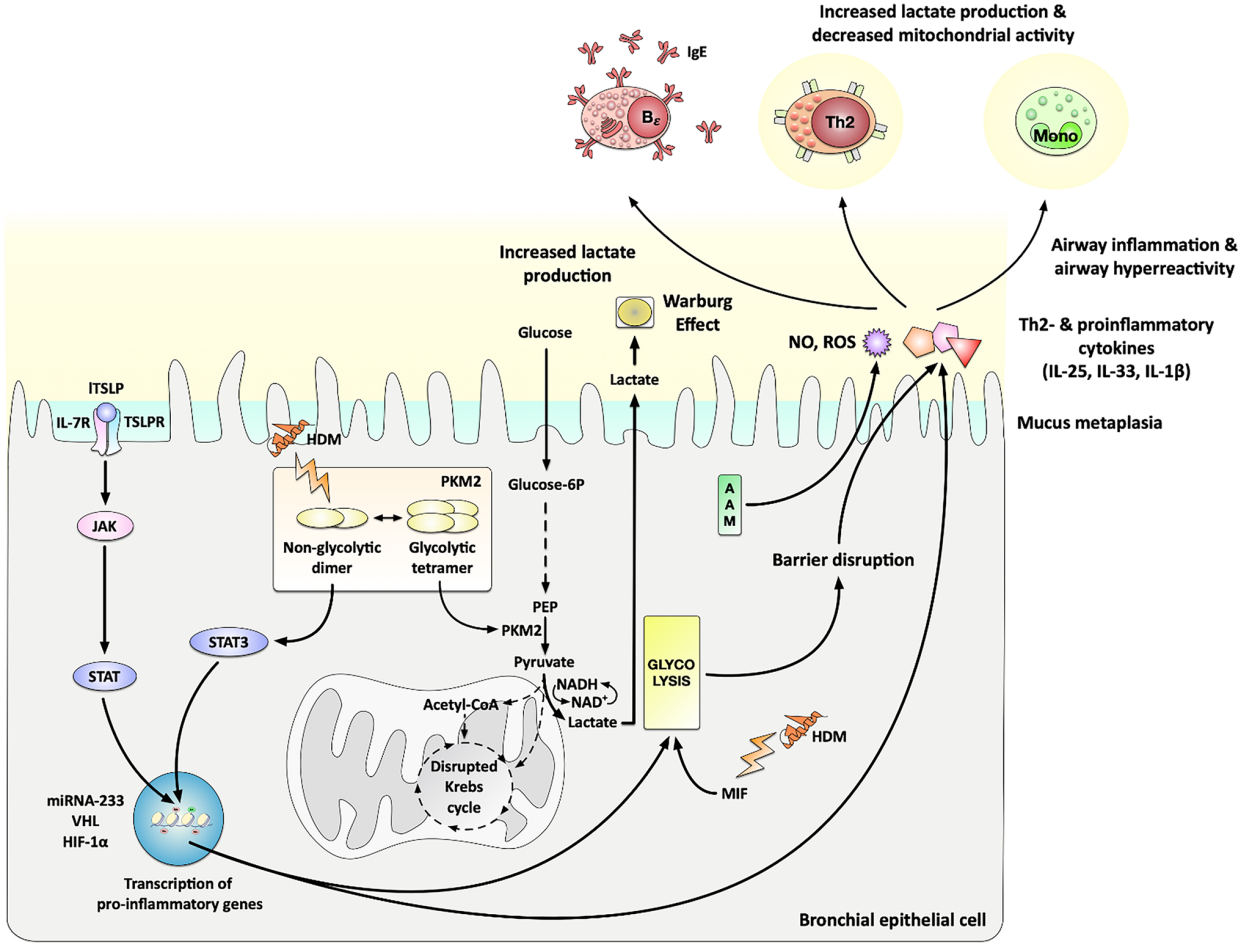
Metabolic adaptions observed in bronchial epithelial cells of asthmatic patients and their contribution to allergic inflammation. Bronchial epithelial cells were repeatedly shown to display increased rates of glycolysis with an increased secretion of lactate, a Warburg Effect, and a disrupted Krebs cycle. Here, the dimeric, non-glycolytic isoform of the enzyme pyruvate kinase isozyme M2 (PKM2, which catalyzes the last step of glycolysis and is an important regulator of overall glycolytic flux) triggered by house dust mite (HDM) exposure, was shown to promote the STAT3-dependent production of pro-inflammatory cytokines. Moreover, the macrophage migration inhibitory factor (MIF), triggered again by HDM exposure, was shown to also promote glycolysis, resulting in barrier disruption and release of pro-inflammatory mediators. Signaling via the long isoform of TSLP binding to the TSLPR/IL-7R-complex was shown to promote allergic inflammation via the JAK/STAT-pathway resulting in the activation of miRNA-233, VHL, and HIF-1α. Also, amino acid metabolism was shown to contribute to the secretion of increased amounts of NO. Inflammatory mediators (NO; ROS; Th2-cytokines and IL-1β) secreted by the bronchial epithelial cells in turn contribute to the activation of other immune cells (e.g., Th2 cells and monocytes, which were also shown to display enhanced rates of glycolysis), the production of IgE antibodies, finally mediating airway inflammation and airway hyperreactivity. For more information see text. Abbreviations: HDM, house dust mite extract; VHL, von Hippel-Lindau; PEP, phosphoenolpyruvate; HIF-1α, hypoxia-inducible factor 1α; JAK, Janus kinase; STAT(3), signal transducer and activator of transcription (3); MIF, macrophage migration inhibitory factor; lTSLP, long isoform of thymic stromal lymphopoietin; TSLPR, TSLP receptor; ROS, reactive oxygen species; NO, nitric oxide

Over the past years, increased rates of glycolysis were reported in bronchial epithelial cells from either obese or asthma patients [48] and glycolytic reprogramming has emerged as a key parameter of allergic airway disease implicated in the development of mucus metaplasia, airway inflammation, and airway hyperreactivity (Fig. 2) [36, 37].

Recently, serum lactate levels were found to be significantly elevated in clinically stable asthmatic patients and chronic obstructive pulmonary disease patients compared to healthy controls. Moreover, CD4^+^ T cells derived from either asthmatics or a mouse model of ragweed asthma produced higher amounts of lactate upon stimulation. Interestingly, lactate stimulated T cell proliferation at low concentrations, whereas at high concentrations, it inhibited proliferation. Inhibiting aerobic glycolysis using dichloroacetate (which inhibits pyruvate dehydrogenase kinase 1 (*PDK1*)) inhibited lactate production, T cell proliferation, production of T cell cytokines (IL-5, IFN-γ IL-17), and airway inflammation while at the same time promoting IL-10-secreting regulatory T cells (Tregs) [50].

Niu et al. showed monocytes from asthmatic patients and lungs from ovalbumin-sensitized and challenged mice to display increased lactate production and enhanced activity of glycolytic enzymes (Fig. 2). This enhanced level of glycolysis was accompanied by decreased ATP production and a reduced activity of mitochondrial respiratory chain complexes I and III, suggesting a switch from OxPhos to aerobic glycolysis in asthma. Interestingly, by acting at upstream regulatory elements and regulating co-factor production, hydrogen (H_2_) was able to reduce airway inflammation by reducing both the activity of glycolytic enzymes and HIF-1α activation while stimulating the activity of OxPhos enzymes [51]. Therefore, hydrogen was able to regulate metabolic programming by reversing the metabolic switch towards aerobic glycolysis.

The establishment and maintenance of asthmatic lung inflammation critically depends on the production of Th2-promoting cytokines such as thymic stromal lymphopoietin (TSLP) [52]. TSLP exists as two distinct isoforms, a long form (lTSLP) and a short form (sTSLP) [53, 54]. Yu et al. found that levels of lTSLP were significantly increased in asthma airway epithelial cells promoting miRNA-233/Von Hippel-Lindau (VHL)/HIF-1α-dependent pro-inflammatory cytokine production and aerobic glycolysis from airway epithelial cells, whereas levels of sTSLP were decreased (Fig. 2). Interestingly, inhibition of glycolysis significantly decreased inflammatory cytokine levels (IL-25 and IL-33), suggesting glycolysis to be involved in asthmatic lung inflammation [55].

In contrast, sTSLP reduced both inflammation of asthmatic airways (reduced airway hyperreactivity, Th2 cytokine production, and IgE production) and aerobic glycolysis in mice by decreasing lactate dehydrogenase A (LDHA) and lactic acid levels in bronchoalveolar lavage fluid (BALF), as well as HIF-1α- and LDHA-protein levels in airway epithelial cells of an asthma mouse model. Mechanistically, both lTSLP and sTSLP induced formation of TSLPR and IL-7R receptor complexes. However, while lTSLP induced phosphorylation of JAK1, JAK2, and STAT5, sTSLP induced little phosphorylation of JAK1 and STAT5. Therefore, it was suggested that sTSLP suppressed inflammation and aerobic glycolysis in asthmatic airway epithelial cells by occupying the binding site of lTSLP in the TSLPR/IL-7R receptor complex [55].

Transforming growth factor beta (TGF-β) was also shown to activate the PI3K-Akt-mTOR pathway in lung myofibroblasts which in turn activates activating transcription factor 4 (ATF4) driving increased expression of the serine-glycine synthesis pathway enzymes that result in excessive deposition of collagen proteins and scarring in idiopathic pulmonary fibrosis. In this context, TGF-β was also shown to increase both glycolysis and mitochondrial oxygen consumption in lung fibroblasts. Here, increased glycolytic flux promoted the conversion of the glycolytic intermediate 3-phosphoglycerate into glycine, the most abundant amino acid found in collagen protein [56].

Macrophage migration inhibitory factor (MIF) was originally described as a T cell-derived protein that could prevent macrophage migration thereby modulating inflammatory responses [39]. Interestingly, MIF overexpression was detected in BALF, serum, and sputum of asthmatic patients where it correlated with the release of pro-inflammatory cytokines TNF-α, IL-1β, and IL-6 [39, 57]. In 16HBE cells, Lan et al. found that house dust mite (HDM) application triggered expression of MIF which in turn promoted epithelial cell barrier disruption and release of Th2-cytokines by accelerating aerobic glycolysis (Fig. 2). Inhibition of MIF *in vivo* reduced airway hyperactivity, airway inflammation, and IgE production in a chronic mouse asthma model [39].

In line with an increased glycolytic metabolism in asthma, Qian et al. showed primary nasal epithelial cells from asthmatic patients to produce more lactate compared to healthy controls and higher lactate levels in asthmatic sputum samples [36].

In a murine model of allergic asthma, the increased rates of glycolytic flux, glucose consumption, expression of glycolysis genes, and lactate production were found to be dependent on IL-1(α/β). Moreover, enhanced glycolysis was shown to be required for HDM-induced Th2 cytokine release from tracheal epithelial cells. Mechanistically, both the inhibition of IκB kinase ε and the increases in the glycolytic enzyme LDHA were required for HDM-induced glycolysis and pathogenesis of allergic airway disease [36].

Pyruvate kinase M2 (PKM2) is the enzyme catalyzing the last step of glycolysis that also acts as an important regulator of overall glycolytic flux. PKM2 exists in two isoforms, a tetrameric isoform that converts phosphoenolpyruvate to pyruvate and a dimeric isoform with alternative non-glycolytic functions enhancing the transcription of several pro-inflammatory cytokines as a transcriptional coactivator (Fig. 2) [58, 59]. In a recent follow-up study, van de Wetering et al. showed the dimeric form of PKM2 to play a crucial role in the pathogenesis of allergic airways disease by increasing signal transducer and activator of transcription 3 (STAT3)-dependent IL-1β–induced pro-inflammatory signaling in a mouse model of HDM-induced asthma (Fig. 2) [37]. Consequently, TEPP46, a small molecule activator of PKM2, which stabilizes the glycolytic tetrameric PKM2 isoform, decreased IL-1β–mediated airway inflammation as well as expression of pro-inflammatory mediators and attenuated airway eosinophils and mucus metaplasia [37].

In a follow-up study, the same authors showed that glutathione-S-transferase P (GSTP) contributes to IL-1β-induced glycolysis and pro-inflammatory signaling in epithelial cells by S-glutathionylation-mediated disruption of the glycolytic activity of the PKM2 tetramer [60]. In line with their findings from the mouse asthma model, GSTP levels were increased in the sputum of severe asthma patients, and sputum proteomics and transcriptomics also showed strong correlations between GSTP, PKM2, and glycolysis, pointing to a putative PKM2-GSTP-glycolysis signature associated with severe asthma [60].

In aggregate, these results suggest enhanced glycolysis to be critically important for the amplification of allergen-induced pro-inflammatory responses and highlight the importance of the GSTP/PKM2-axis in regulating this process, suggesting GSTP/PKM2 as a novel potential target for the development of IL-1β-associated, glycolysis in asthma [37, 60].

Their results were independently confirmed by Manuel et al., who showed HDM to trigger PKM2-dependent glycolytic reprogramming and airway inflammation in a murine model of obese allergic asthma and airway epithelial cells [38]. These results suggest alternative, non-glycolytic functions of PKM2 to exert pro-inflammatory roles in asthma by glutathione-dependent protein oxidation via a putative IFN-γ–glutaredoxin 1 pathway [38].

Besides increased glycolysis, fatty acid metabolism was repeatedly described to be altered in asthmatic patients [61, 62]. Over the last years, several studies compared asthmatics to healthy controls using either metabolomics or transcriptomics approaches.

Zhu et al. performed an unbiased metabolomics approach with BALF samples of 12 patients undergoing an acute inflammatory response that was experimentally induced by bronchial allergen challenge [63]. Interestingly, unbiased associations of 549 metabolites changed by allergen challenge identified two distinct metabolic profiles in a group of allergic asthmatics that were otherwise indistinguishable under basal conditions [63]. Here, among other pathways relevant to asthma, especially an upregulation of saturated fatty acid synthesis and mitochondrial beta-oxidation of saturated fatty acids was observed upon allergen challenge [63].

Johnson et al. quantified lipid mediators in the nasal airway epithelium in three non-atopic controls, four mild to moderate asthmatics, and four severe asthmatics by LC–MS [64]. In line with an increase in fatty acid metabolism in asthmatics, they found lower levels of pro-resolving 15-HEPE, 19,20-DiHDPA, RvD5, 14-HDHA, 17-HDHA, and 13-HOTrE. In contrast, levels of mainly pro-inflammatory prostaglandin E2 were increased in asthmatic samples, suggesting the levels of lipid mediators in nasal epithelium to distinguish asthma patients form healthy controls [64].

Eosinophils are critical for the local production of cys-leukotrienes in asthma [65]. In line with the higher production of the pro-inflammatory lipid mediator prostaglandin E2 observed by Johnson et al., Miyata et al. reported nasal polyp-derived eosinophils from patients with eosinophilic rhinosinusitis to present a characteristic fatty acid metabolism with selectively higher production of leukotriene D4 [66].

Confirming higher rates of fatty acid metabolism in asthmatics, bronchial smooth muscle (BSM) from asthmatic patients was characterized by a switch towards increased fatty acid consumption and increased rates of mitochondrial respiration [67]. In line with these results, the authors observed a significant decrease in arachidonic-, stearic-, and linoleic acid content in asthmatic BSM cells compared to non-asthmatic control cells. Interestingly, in this study, no differences in glucose uptake were observed between asthmatic and non-asthmatic BSM cells [67]. Esteves et al. also found increased levels of two molecules involved in the internalization of fatty acids, carnitine palmitoyl transferase-2 (CPT2) and LDL-receptor, in asthmatic BSM cells [67]. Here, blocking of either CPT2 or LDL-receptor strongly reduced BSM cell proliferation, confirming the reliance of asthmatic, hyperproliferative BSM cells on increased FAO and suggesting fatty acid metabolism as a novel treatment target.

Ravi et al. performed unbiased transcriptome analyses of bronchial epithelial cells (BECs) from either mild, moderate, or severe asthma patients to investigate metabolic adaptations in asthma [68]. Patients with severe asthma displayed a reduction in OxPhos genes, while for patients with mild asthma, this reduction was less pronounced and more heterogeneous [68]. In contrast to this, both genes related to fatty acid metabolism and certain lipid species (PCs, LPCs, lysophosphatidylethanolamines, and BMP) were significantly upregulated in BECs from all asthma patients independently of asthma severity, clearly differentiating asthma patients from healthy controls and demonstrating a persistently altered fatty acid metabolism in asthmatic bronchial epithelium [68]. This metabolic shift possibly affecting purine metabolism, amino acid biosynthesis, and glycolysis was reversed in patients who underwent asthma treatment with bronchial thermoplasty, resulting in a gene expression profile more comparable to healthy controls [68].

In a mechanistic study, Lechner et al. described macrophages derived either from asthma patients, HDM-sensitized mice, or macrophages trained with HDM extract *in vitro* to produce high amounts of CCL17 and cysLTs, both key mediators involved in asthma while not generally increasing the production of pro-inflammatory cytokines [69]. This allergen-driven trained immunity program depended on formyl peptide receptor 2 (FPR2)- and TNF-signaling and resulted in metabolic reprogramming and lysine demethylase (KDM) 1A–mediated histone demethylation (particularly H3K4 and H3K9) and the potential removal of repressive marks to enhance Th2 responses [69]. Therefore, this trained immunity response may contribute to both chronification and exacerbation of allergic asthma and could be a promising target for preventing asthma.

Polyunsaturated fatty acids (PUFAs) were repeatedly shown to have health-promoting effects [70, 71]. Stearoyl-coenzyme A desaturase (SCD) is the rate-limiting enzyme in the synthesis of PUFAs [72]. Impaired fatty acid metabolism was suggested to contribute to asthma pathology [43, 73, 74]. Rodriguez-Perez et al. observed significantly reduced serum fatty acid levels (palmitoleic acid, arachidonic acid, and docosahexaenoic acid) and a suppressed polyunsaturated:saturated fatty acid ratio in serum of non-obese asthma patients, suggesting reduced desaturase activity in non-obese asthma patients with severe disease [62]. Their results were confirmed in human bronchial epithelial cells and both mouse OVA and HDM models where animals also showed both reduced desaturase activity and reduced SCD expression in the lung [62]). Here, inhibition of SCD promoted airway hyper-responsiveness in mice and reduced anti-viral defense in bronchial epithelial cells [62]. Taken together, their results suggested that SCD gene expression and therefore, the levels of potentially anti-inflammatory PUFAs may be regulated by inflammatory responses in the lung. Therefore, SCD may be a promising target for the therapy of asthma.

Besides glucose and fatty acid metabolism, amino acid metabolism was also reported to be altered in asthma. Arginine metabolism in particular was identified as a key driver of Th2 airway inflammation [75] and to affect the development of asthma [75–77]. Arginine is a semi-essential amino acid that is metabolized to generate NO by several enzymes, including the cytosolic inducible nitric oxide synthase (iNOS), the mitochondrial arginase-2 (ARG2), and ARG1 which mostly expressed in the cytosol of hepatic cells [78, 79]. ARG2 gene variants are among the first and most consistently found single nucleotide polymorphisms (SNPs) associated with asthma in genome-wide association studies (GWAS) [80–82].

In line with these results, expressions of iNOS and ARG1/2 were found to be upregulated in asthmatic patients, leading to higher levels of exhaled NO and more severe symptoms (Fig. 2) [75–77]. Interestingly, patients with high levels of exhaled NO were reported to also have increases in airway lactate levels, suggesting a link between enhanced levels of glycolysis, arginine metabolism, and exhaled NO in some asthma phenotypes [77].

In this context, Asosingh et al. showed arginine metabolism to be a critical modulator of the severity of inflammation and remodeling in eosinophilic and neutrophilic asthma [83]. In their asthma cohort, ARG2 variants associated with lower arginase activity, combined with high levels of fractional exhaled nitric oxide identified a more severe asthma phenotype [83]. In line with these results, ARG2 knock-out mice had the highest levels of airway inflammation, airway remodeling, and lung inflammatory cytokines, suggesting a central role for arginine metabolism in the control of lung inflammation. In line with their results, deletion of ARG2 was shown to increase HIF- and STAT6-signaling resulting in increased eosinophilic Th2 inflammation and Th2 cytokine production [75].

In an independent study, Zhou et al. identified decreased levels of L-citrulline and L-arginine in the serum of 30 young patients with chronic persistent asthma [84], suggesting the balance in arginine metabolism to be important for the pathogenesis and progression of chronic asthma. They suggested that therapeutic supplementation of L-citrulline could restore airway NO production and therefore reduce inflammation [84].

Using ultra-high performance liquid chromatography–mass spectrometry (UHPLC-MS), Liu et al. characterized metabolic profiles in induced sputum samples from both healthy controls and asthmatic patients [85]. They found different inflammatory asthma phenotypes to have specific metabolic profiles with 77 differential metabolic signatures which belonged to five underlying pathways (histidine metabolism, glycerophospholipid metabolism, nicotinate and nicotinamide metabolism, and linoleic acid metabolism as well as phenylalanine-, tyrosine-, and tryptophan-biosynthesis) [85]. Here, levels of adenosine 5′-monophosphate, allantoin, and nicotinamide in sputum samples predicted rate ratios of severe asthma exacerbations [85].

Recently, Bravo-Solarte et al., using bronchial brush samples, described genomic evidence of misregulated glutamine metabolism in airway epithelial cells from asthma patients [86]. Based on a secondary analysis of two open-access microarray datasets from asthmatics and healthy controls, the authors found upregulation of enzymes involved in the glutamine metabolism including glutamic‐oxaloacetic transaminase 1, glutamic‐oxaloacetic transaminase 2, nitric oxide synthase 2, glutathione peroxidase 7, phosphoserine aminotransferase 1, pyrroline‐5‐carboxylate reductase 1, sirtuin 7, solute carrier family 7 member 11, and solute carrier family 39 member 8 [86].

Chiu et al. performed a metabolome analysis of plasma and urine samples using 1H-NMR spectroscopy comparing 28 asthmatic children to 26 age-matched controls [87]. They found significantly higher plasma histidine levels, paralleled by lower urinary 1-methylnicotinamide and trimethylamine N-oxide (TMAO) levels in children with asthma compared to healthy controls [87]. Moreover, plasma pyruvate and urine valine, leucine, and isoleucine degradation-metabolism were significantly associated with allergic sensitization for childhood asthma [87]. Therefore, the authors suggested that plasma pyruvate metabolism, via the production of acetyl-CoA, may play a role in serum IgE production whereas branched-chain amino acid metabolism in urine primarily reflects food allergic reactions [87].

In a follow-up study, the authors combined blood 1H-NMR metabolomic analyses with airway microbiome composition analyses based on 16S rRNA sequencing in 15 lowly sensitized non-atopic asthma and 13 highly sensitized atopic asthma patients [88]. Here, four metabolites (tyrosine, isovalerate, glycine, and histidine) were uniquely associated with lowly sensitized asthma, whereas acetic acid was uniquely associated with highly sensitized asthma [88]. Branched short-chain fatty acids are generated by bacteria catabolizing the amino acids valine, isoleucine, and leucine [88]. The authors furthermore found levels of the microbial-derived metabolites isobutyric and isovaleric acid to be strongly associated with highly and lowly sensitized asthma, respectively [88]. Moreover, glutamine levels were strongly associated with IgE levels and paralleled by a reduced abundance of the genus *Prevotella* of the phylum *Bacteriodetes*. In addition, *Atopobium* *spp*. were both strongly correlated with acetic acid and glutamine [88]. These results suggested that microbial-derived branched short-chain fatty acids may participate in the molecular immune responses contributing to different endotypes of asthma.

As complex as asthma with its different phenotypes is, as complex seem to be the metabolic changes associated with the establishment and maintenance of asthmatic lung inflammation. The currently available literature points to substantial metabolic changes in glucose, fatty acid, and amino acid metabolism in asthmatic patients. Here, the specific details seem to depend on the investigated patient collective and asthma phenotype as well as disease severity (see above).

### Food Allergy

De Paepe et al. recently reviewed metabolic alterations in food allergic children. By constructing a food allergy dataset from previous studies, 277 potential biomarkers in human and animal biofluids were investigated with regard to their contribution to the development of food allergy [89]. Here, due to changes in plasma levels of tryptophan in food allergic patients, decreased indoleamine 2,3-dioxygenase-1 (IDO-1) levels were proposed [89]. Moreover, altered sphingolipid and histidine metabolism were observed [89]. Reduced levels of short chain fatty acids in human feces led to a metabolic switch towards aerobic glycolysis, shifting the immune response towards Th2-responses [89].

Crestani et al. compared serum samples of children with either food allergy alone, asthma alone, or children with both food allergy and asthma using mass spectrometry-based untargeted metabolomic profiling [90]. Food allergy patients showed distinct alterations in lipid metabolites, the most prominent of them being a decrease in sphingolipid levels, including sphingomyelins and ceramides [90]. Sphingolipids can either be derived from dietary sources or produced by bacteroides species in the gut microbiome [91], suggesting changes in the gut microbiome to be responsible for the observed differences. Interestingly, children with both asthma and food allergy displayed metabolomic profiles that aligned with food allergy alone but not asthma [90].

Therefore, the currently limited studies suggest primarily lipid and amino acid metabolism to be altered in food allergy. However, more studies both in humans and in-depth analyses using mouse models are needed to better understand the observed changes.

### Atopic Dermatitis

Goleva et al. performed mass spectrometry-based proteomics analysis of skin tape strip samples of non-lesional skin of 62 children with atopic dermatitis (with and without food allergy) and non-atopic controls [92]. They identified three functional groups of proteins to be altered in non-lesional skin of atopic dermatitis patients with food allergy: (I) keratin intermediate filaments, (II) proteins associated with inflammatory responses (S100 proteins, alarmins, protease inhibitors), and (III) glycolysis and oxidative stress response proteins (glycolytic enzymes, oxidative stress response enzymes) [92]. The authors suggested these changes to be a compensatory mechanism to counterbalance the initial tissue reorganization associated with Th2 responses in the AD skin.

## Conclusion

Over the last years, a lot of studies have started to analyze the immune-metabolic phenotype of both allergic patients as well as the distinct cell types contributing to the establishment and maintenance of allergic sensitization. So far, the immune-metabolic changes observed in allergy are complex and depend on the investigated disease and cell type (for more details see part 2: cell type-specific findings).

In terms of allergic diseases, currently, most studies focus on asthma. Initial results suggested that asthma is associated with a shift in cellular metabolism towards Warburg metabolism. While these results were meanwhile independently confirmed many times, the currently available literature also points to substantial changes in fatty acid- and amino acid metabolism in asthmatic patients (depending on investigated patient collective, asthma phenotype, and disease severity), making the overall picture more complex. For other allergic diseases (e.g., food allergy or atopic dermatitis), there are currently too little studies to draw meaningful overall conclusions. Here, definitely further studies, including both the analysis of allergic patients and mechanistic animal models, are required.

These distinct immune-metabolic changes observed in allergic patients have strongly suggested that the extensive crosstalk between cellular metabolism and immune cell effector function holds potential as a therapeutic target to improve disease treatment. In the second part of this review series, we will discuss the recent discoveries concerning metabolic changes associated with the activation of the major cell types involved in allergic reactions and report on the initial application of these discoveries in allergy treatment.

## Data Availability

Data files used to generate the figures shown in this paper are available from the corresponding author upon request.

## Abbreviations

AAM: Amino acid metabolism
APC: Antigen-presenting cell
ARG1/2: Arginase-1/2
ATF4: Activating transcription factor 4
BALF: Bronchoalveolar lavage fluid
BECs: Bronchial epithelial cells
BSM: Bronchial smooth muscle
DC: Dendritic cell
ETC: Electron transport chain
FAO: Fatty acid oxidation
FPR2: Formyl peptide receptor 2
GSTP: Glutathione-S-transferase P
GWAS: Genome-wide association studies
H_2_: Hydrogen
HDM: House dust mite
HIF-1α: Hypoxia inducible factor 1 subunit alpha
IDH: Isocitrate dehydrogenase
IDO-1: Indoleamine 2,3-dioxygenase-1
iNOS: Inducible nitric oxide synthase
KDM: Lysine demethylase
LDHA: Lactate dehydrogenase A
MIF: Macrophage migration inhibitory factor
mTOR: Mammalian target of rapamycin
NO: Nitric oxide
OxPhos: Oxidative phosphorylation
PDK1: Pyruvate dehydrogenase kinase 1
PKM2: Pyruvate kinase isoenzyme M2
PUFA: Polyunsaturated fatty acid
ROS: Reactive oxygen species
SCD: Stearoyl-coenzyme A desaturase
SNPs: Single nucleotide polymorphisms
STAT(-1/3): Signal transducers and activators of transcription 1/3
TGF-β: Transforming growth factor beta
TMAO: Trimethylamine N-oxide
TSLP: Thymic stromal lymphopoietin
Treg: Regulatory T cell
(l/s)TSLP: (Long/ short form of) thymic stromal lymphopoietin
UHPLC-MS: Ultra-high performance liquid chromatography–mass spectrometry
VHL: Von Hippel-Lindau
